# Retraction Note: Comparative analysis of laparoscopic choledocholithiasis and ERCP treatment after cholecystectomy

**DOI:** 10.1186/s12893-025-02902-z

**Published:** 2025-04-16

**Authors:** Jun Zhang, Liqiang Li, You Jiang, Wenbo Li, Liang Li

**Affiliations:** 1 Department of General Surgery, the Second People’s Hospital of Hefei or Hefei Hospital Affiliated to Anhui medical University, Hefei, 230011 China; 2 Second People ’ s Hospital, Bengbu Medical College, Hefei, 230011 China) China

**Retraction Note: BMC Surgery (2023) 23:304**

10.1186/s12893-023-02207-z

The Editor has retracted this article because the authors have not provided documents confirming that ethics approval was obtained prior to the commencement of the study.

The authors disagree with this retraction.

